# Subnanometer cobalt oxide clusters as selective low temperature oxidative dehydrogenation catalysts

**DOI:** 10.1038/s41467-019-08819-5

**Published:** 2019-02-27

**Authors:** Sungsik Lee, Avik Halder, Glen A. Ferguson, Sönke Seifert, Randall E. Winans, Detre Teschner, Robert Schlögl, Vasiliki Papaefthimiou, Jeffrey Greeley, Larry A. Curtiss, Stefan Vajda

**Affiliations:** 1 X-ray Science Division, Argonne National Laboratory, 9700 South Cass Avenue, Lemont, IL 60439 USA; 2 Materials Science Division, Argonne National Laboratory, 9700 South Cass Avenue, Lemont, IL 60439 USA; 3 Fritz-Haber-Institut der Max-Planck Gesellschaft, Faradayweg 4-6, 14195 Berlin, Germany; 4 Max-Planck-Institute for Chemical Energy Conversion, Stiftstrasse 34-36, 45470 Mülheim an der Ruhr, Germany; 5 ICPEES, Institut de Chimie et des Procédés pour l’Energie, l’Environnement et la Santé, University of Strasbourg, 25 rue Becquerel, 67087 Strasbourg, France; 6 School of Chemical Engineering, Purdue University, 480W Stadium Mall Drive, West Lafayette, IN 47907 USA; 7 Institute for Molecular Engineering, University of Chicago, Eckhardt, 5640 South Ellis Avenue, Chicago, IL 60637 USA

## Abstract

The discovery of more efficient, economical, and selective catalysts for oxidative dehydrogenation is of immense economic importance. However, the temperatures required for this reaction are typically high, often exceeding 400 °C. Herein, we report the discovery of subnanometer sized cobalt oxide clusters for oxidative dehydrogenation of cyclohexane that are active at lower temperatures than reported catalysts, while they can also eliminate the combustion channel. These results found for the two cluster sizes suggest other subnanometer size (CoO)_x_ clusters will also be active at low temperatures. The high activity of the cobalt clusters can be understood on the basis of density functional studies that reveal highly active under-coordinated cobalt atoms in the clusters and show that the oxidized nature of the clusters substantially decreases the binding energy of the cyclohexene species which desorb from the cluster at low temperature.

## Abstract (web summary)

Current oxidative dehydrogenation processes are based on petroleum cracking that is indirect, environmentally unfriendly, and energy intensive. Here, the authors discover that subnanometer sized cobalt oxide clusters are active for oxidative dehydrogenation of cyclohexane at lower temperatures than reported catalysts.

## Introduction

Exothermic oxidative dehydrogenation of alkanes is an attractive alternative to the energy demanding endothermic dehydrogenation route. However, despite decades of research efforts, the current oxidative dehydrogenation catalysts have limited activity and/or poor selectivity^1^. Supported small metal clusters have been shown to possess distinct catalytic properties not observed in their bulk analogs^2–10^. The special reactivity of the small clusters is believed to come from the unique electronic structure characteristics of the clusters^11–14^. For example, subnanometer Pt clusters have been identified as a highly active, as well as highly selective catalyst for the oxidative dehydrogenation of propane; the study provides a molecular level understanding of the catalyst^8^.

**Fig. 1:**
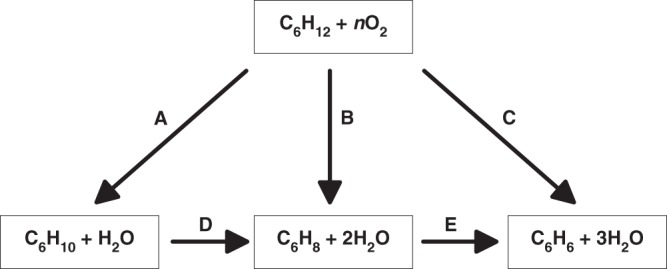
Simplified reaction scheme of the ODH of cyclohexane

The oxidative dehydrogenation (ODH) of alkanes is exothermic overall and is, thus, a desirable substitute to dehydrogenation, which is an endothermic process requiring significant energy input. Current ODH processes are based on petroleum cracking that is indirect, environmentally unfriendly, and energy expensive due to the high temperatures required^15,16^. Thus, discovery of more efficient and direct catalysts for ODH is of immense economic importance. In addition, one must find catalysts that can perform partial dehydrogenation. For example, in the case of cyclohexane ODH as shown in Fig. 1, it is challenging to find catalysts that do not dehydrogenate products all the way to benzene.

Co-based catalysts have recently been getting lots of attention in dehydrogenation reactions, for e.g. few-atom Co(II) ions doped Zr-based MOFS NU-1000 was shown to be active around 230 °C for oxidative dehydrogenation of propane^17,18^; Co catalysts in other forms have also been used for oxidative dehydrogenation reactions, including highly dispersed CoOx in layered double oxides^19^, or cobalt-decorated graphene shells for both dehydrogenation and hydrogenation reactions^20^. Herein, we report results on subnanometer cobalt oxide clusters for the oxidative dehydrogenation of cyclohexane. The results show that under oxidative conditions oxidized subnanometer 4- and 27- atom cobalt clusters (Co_4_, Co_27_) are active at as low as 100 °C in cyclohexane oxidative dehydrogenation. In addition, product selectivity can be altered by changing the reaction conditions, in this case the oxygen to cyclohexane ratio.

This dramatic lowering of the temperature can be understood on the basis of density functional studies, which indicate that the oxidized nature of the Co clusters substantially decreases the binding energy of the alkene product, i.e. potential poisoning of the catalyst, in comparison with their metallic counterparts. The efficacy of sub-nanometer cobalt clusters for oxidative dehydrogenation at low temperatures is important since cobalt would be very attractive in a variety of industrially important oxidative processes that often utilize precious metals as catalysts and require high temperatures^21–23^. Such developments would have practical implications ranging from more energy efficient and environmentally friendly strategies for chemical synthesis to the replacement of current petrochemical feedstocks by inexpensive abundant small alkanes.

## Results

### Catalytic performance under atmospheric pressure

**Fig. 2:**
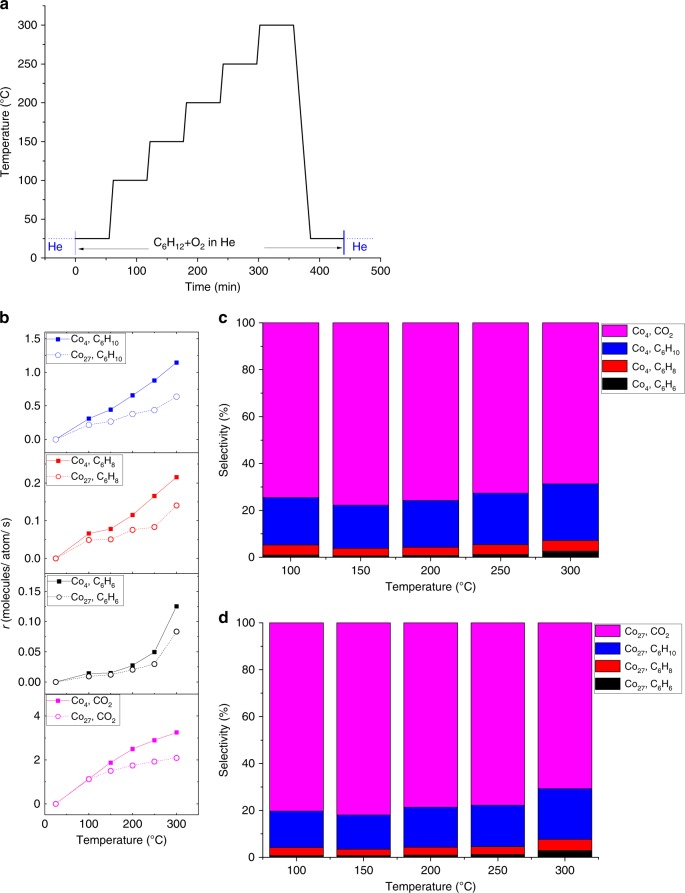
Performance of alumina-supported Co clusters under oxygen rich conditions (C_6_H_12_:O_2_ = 1:10). **a** Temperature ramp with gas environment applied. **b** *r* (rate of formation) for the major products cyclohexene (m/z = 67) on the Co_4_/Al_2_O_3_ catalyst (blue squares) and Co_27_/Al_2_O_3_ catalyst (blue circles), cyclohexadiene (m/z = 79) on the Co_4_/Al_2_O_3_ catalyst (red squares) and Co_27_/Al_2_O_3_ catalyst (red circles), and benzene (m/z = 78) on Co_4_/Al_2_O_3_ catalyst (black squares) and Co_27_/Al_2_O_3_ catalyst (black circles), and the byproduct CO_2_ (m/z = 44) on the Co_4_/Al_2_O_3_ catalyst (pink squares) and Co_27_/Al_2_O_3_ catalyst (pink circles). Cyclohexadiene mass is a superposition from 1,3- and 1,4- cyclohexadiene (both isomers have same electron ionization cross section^53^). **c** Carbon-based selectivity for the dominant reaction products with Co_4_/Al_2_O_3_ catalyst (blue: cyclohexene, red: cyclohexadiene, black: benzene, pink: CO_2_), and **d** Carbon-based selectivity for the dominant reaction products for Co_27_/Al_2_O_3_ catalyst (blue: cyclohexene, red: cyclohexadiene, black: benzene, pink: CO_2_). The interconnecting lines serve as guide to the eye

In this work, positively charged cobalt clusters were produced in a cluster beam (see Supplementary Figure 1 for a typical mass spectrum) and using a mass spectrometer, the Co_4_ or Co_27_ clusters were filtered out and deposited at 0.1 atomic monolayer coverage on a ~3 ML thick alumina film prepared by atomic layer deposition on the top of a doped Si chip. Applying the temperature ramp and experimental conditions shown in Fig. 2a, combined in situ grazing incidence small angle X-ray scattering (GISAXS), grazing incidence X-ray absorption near-edge structure (GIXANES) and temperature programmed reaction (TPRx) experiment under a total pressure of 800 mbar was used to monitor the sintering resistance, oxidation state and catalytic performance of the clusters.

**Fig. 3:**
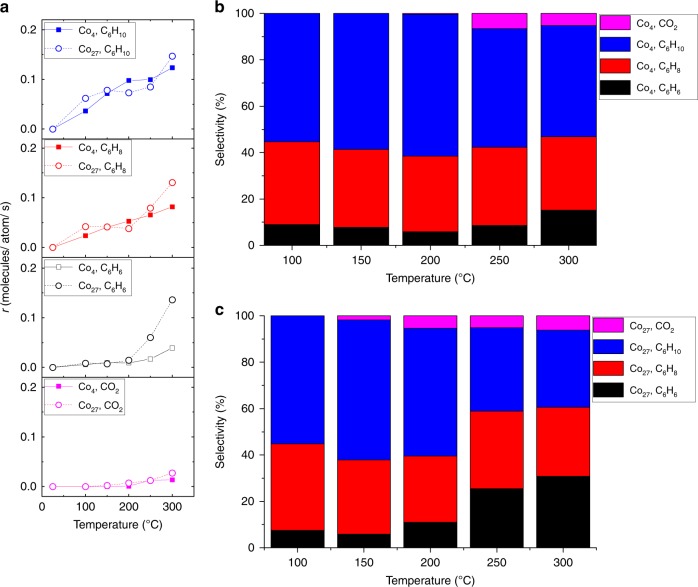
Performance of alumina-supported Co clusters under oxygen lean conditions (C_6_H_12_:O_2_ = 10:1). **a** *r* (rate of formation) for the major products cyclohexene (m/z = 67) on the Co_4_/Al_2_O_3_ catalyst (blue squares) and Co_27_/Al_2_O_3_ catalyst (blue circles), cyclohexadiene (m/z = 79) on the Co_4_/Al_2_O_3_ catalyst (red squares) and Co_27_/Al_2_O_3_ catalyst (red circles), benzene (m/z = 78) on Co_4_/Al_2_O_3_ catalyst (black squares) and Co_27_/Al_2_O_3_ catalyst (black circles), and the byproduct CO_2_ (m/z = 44) on the Co_4_/Al_2_O_3_ catalyst (pink squares) and Co_27_/Al_2_O_3_ catalyst (pink circles). Cyclohexadiene mass is a superposition from 1,3- and 1,4- cyclohexadiene (both isomers have same electron ionization cross section^53^). **b** Carbon-based selectivity for the dominant reaction products with Co_4_/Al_2_O_3_ catalyst (blue: cyclohexene, red: cyclohexadiene, black: benzene, pink: CO_2_), and **c** Carbon-based selectivity for the dominant reaction products for Co_27_/Al_2_O_3_ catalyst (blue: cyclohexene, red: cyclohexadiene, black: benzene, pink: CO_2_). The interconnecting lines serve as guide to the eye

The experiments were performed using two different gas mixture compositions of cyclohexane (C_6_H_12_) and oxygen (O_2_) at 1:10 and 10:1 ratio of cyclohexane to oxygen ratio, both seeded in helium. Benzene, cyclohexene, cyclohexadiene, and CO_2_ were identified as the major products, along with trace amounts of cyclohexanone and cyclohexanol present. Under identical reaction conditions, the blank ALD alumina support showed no activity. Figure 2b shows the per total Co atom rate of formation (*r*) of the main products cyclohexene (C_6_H_10_), cyclohexadiene (C_6_H_8_), benzene (C_6_H_6_) and carbon dioxide (CO_2_) formed in the 1:10 C_6_H_12_/O_2_ mixture on the 4- and 27-atom cobalt clusters. The samples are active  at 100 °C, with the Co_4_ clusters possessing a higher *r* than the Co_27_ clusters. *r* is calculated by the total number of product molecules formed per Co atom in the catalyst per second. As seen from the selectivity plots in Fig. 2c, d, the selectivities are very similar for the two cluster sizes, with combustion being the dominant and almost no benzene produced. The results indicate a highly efficient activation of molecular oxygen by the subnanometer Co clusters, demonstrated by the high amount of CO_2_ produced already at 100 °C. The reactivity results for the 10:1 C_6_H_12_/O_2_ mixture are shown in Fig. 3. *r*-values are tabulated in Supplementary Table 2. A comparison of performance of the subnanometer cobalt clusters with other reported cyclohexane ODH catalysts is listed in Supplementary Table 3, showing excellent performance of the subnanometer clusters. Measurable activity is again found at 100 °C, with the formation of CO_2_ apparently suppressed on both cluster sizes, with no CO_2_ observed on the Co_4_ clusters up to 200 °C. However, we need to note two important facts. First, there is an about order of magnitude difference observed in the total reaction rate for the two gas compositions; thus the lower conversion under oxygen lean conditions can shift selectivity towards the C6 products. Second, the first true measurement point is at 100 °C (no plateau in activity seen), thus a possible activity at lower temperatures is neglected. The activation energies estimated from the rates of product formation are 12.1 ± 0.9 kJ mol^−1^ (0.13 ± 0.01 eV), 24.4 ± 0.4 kJ mol^−1^ (0.25 ± 0.00 eV), and 32.0 ± 3.2 kJ mol^−1^ (0.33 ± 0.03 eV) for cyclohexene, cyclohexadiene, and benzene, respectively (as calculated from the Arrhenius plots shown in Supplementary Figures 9; ± denotes standard error). It is worth noting the very similar trends in activity and selectivity of Co_4_ and Co_27_. Another interesting feature, at comparable per atom activities, is the apparently worse linearity of the data in the Arrhenius plots for the larger clusters. We hypothesize, that it may reflect a higher fluxionality/structural dynamics of the larger clusters leading to more pronounced changes in their ensemble’s average structure and related activity evolving with temperature, than for the ensembles of smaller clusters, though size effects in cluster-support interactions may play a role as well^24^. The production of all C_6_ products was confirmed in the experiments performed in 1 mbar (L-edge XAS experiments; see Supplementary Table 1 in Supplementary Information), though a direct comparison of the obtained rates of formation cannot be made due to different reaction conditions.

The main findings are the dramatically reduced temperature in comparison with previously reported ODH catalyst for propane and cyclohexane and the suppression of CO_2_ production for the lean conditions. The temperature of 100 °C, at which the ODH activity sets off, is lower than reported for propane ODH with Pt clusters (~400 °C)^8^ or in the ODH of cyclohexane by various catalysts, such as VOx (~400 °C)^25^, zeolites doped with transition metals (>300 °C)^26^, copper oxide-based (~250 °C)^27^, Au/Pd-based (200 °C)^23^, or FeO_*x*_, Au/Fe_3_O_4_ and Co_3_O_4_-based catalysts (~200–250 °C)^28,29^. In comparison with per exposed surface Co atom rates obtained for 6 nm Co_3_O_4_ particles^29^ tested under identical reaction conditions, the rate of formation of cyclohexene, cyclohexadiene, and benzene measured on the Co_4_ and Co_27_ clusters are higher by about a factor of 20, 100, and 1.2, respectively.

### Cobalt oxidation state under atmospheric pressure

**Fig. 4:**
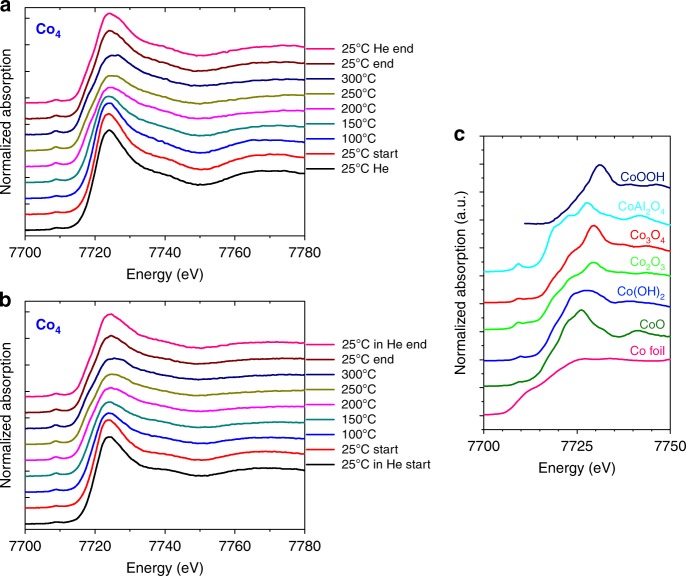
In situ XANES spectra of Co_4_ clusters collected during the applied ramp. **a** spectra acquired under cyclohexane oxygen 1:10 ratio and **b** under and 10:1 ratio seeded in He; spectra collected at the indicated temperatures applied from the start (bottom) of the temperature ramp to its end (top). He denotes spectra collected under helium. **c** XANES spectra of bulk Co standards: Co foil (pink line), CoO (dark green line), Co(OH)_2_ (dark blue line), Co_2_O_3_ (light green line), Co_3_O_4_ (red line), CoAl_2_O_3_ (cyan line) and CoOOH (black line). (Spectrum of CoOOH adapted with permission from ref. ^54^, CopyRight (2018) of the American Chemical Society

The GISAXS data showed no evidence of agglomeration of the clusters during the course of the reaction. The normalized in situ XANES Co-K edge spectra of the Co_4_ clusters obtained for oxygen rich and lean conditions are shown in Fig. 4 along with the XANES spectra of bulk Co standards. The features of the spectra of the clusters and their evolution with temperature are very similar under both gas mixtures. When compared with the spectra of the bulk standards Co, CoO, Co_2_O_3_, Co_3_O_4_, CoOOH, Co(OH)_2_, and CoAl_2_O_4_, the spectra of the clusters are indicative of Co(II). There is no evidence of Co(III) from the XANES spectra. The spectra of the as-prepared samples are broad and without distinct features, suggesting that Co clusters occupy various sites on the amorphous substrates, while structural, electronic, and charge effects in subnanometer clusters may be reflected in their spectra as well^30^. The lower intensity of the shoulder around 7719 eV typical of the tetrahedral coordination indicates a small fraction of Co ions at tetrahedral sites^31,32^. The XANES spectra of the cluster samples were analyzed by a linear combination fitting (LCF) using XANES spectra of bulk Co standards are shown in Supplementary Figure 2. (See Supplementary Figures 2b-c and 3b-c in the Supplementary Information showing the results from linear combination fit analysis for Co_4_ clusters.) The results of the analysis for the Co_27_ clusters are shown in Supplementary Figures 2d-e and 3d-e, respectively; examples of fitting results are presented in Supplementary Figures 4 and 5 of the Supplementary Information. The comparison of the spectra and the LCF results show similar trends for both cluster sizes under each reaction mixture and a somewhat higher hydroxide fraction for the 10:1 C_6_H_12_/O_2_ mixture. At room temperature, under He as well as under reactant gas mixture, the Co_4_ and Co_27_ clusters are present in the CoO phase. The features of the XANES spectra of both clusters Co_4_ and Co_27_ start to visibly evolve at 150 °C, and LCF analysis reveals the emergence of a Co(OH)_2_ phase that peaks at the highest temperatures applied (200–300 °C). Since, the fraction of the cobalt hydroxide component is higher under oxygen lean conditions when less water is produced, this hints towards the dehydrogenation of the cyclohexane molecule which causes the hydroxylation of cobalt, rather than the reaction with water formed during the ODH process. This hypothesis is confirmed by the observation that at room temperature, the fraction of the hydroxide is lower—both before and after the applied temperature ramp. The occurrence of a small fraction of CoAl_2_O_4_ at the highest temperatures most likely reflects the evolution of cluster-support interactions during the course of the reaction, possibly accompanied with structural changes in the clusters. Overall, the Co atoms within the clusters retain their oxidation state of 2+ during the entire test. We hypothesize that the reluctance of subnanometer Co clusters to change their 2+ oxidation state may be responsible for their excellent performance as ODH catalysts working at surprisingly low temperatures, through the nanoeffect. This hypothesis is supported by reports on the strongly size-dependent redox behavior of cobalt oxide particles. It was shown, that, for example, wurztite type CoO nanoparticles resist to reduction and oxidation, in contrary to larger oxide particles or macroscopic Co samples^33^. Other studies demonstrated the stable 2+ state of cobalt in subnanometer cobalt oxide clusters when exposed to elevated temperatures^34^ or under cyclohexene ODH conditions^35,36^. Interrogations of oxidized size-selected cobalt nanoparticles, under similar cyclohexane ODH conditions as applied in the present study, determined ~3 nm as the critical size for cobalt oxide particles^37^: Particles larger than 3 nm underwent size-dependent changes in their morphology as well as in the oxidation state of cobalt. In contrary, the smaller particles were robust and retained oxidation state 2+. Such tunability of the oxidation state through variable particle size may offer high-fidelity control knob over the performance of catalysts in general.

### Cobalt oxidation state under low pressure

Additional characterization was performed at the Co-L edge under ultra-high vacuum (UHV) and in situ conditions in 1 mbar pressure (see Supplementary Figure 6 in the Supplementary Information). The XAS characterization reveals that the clusters, independent of their size (4 vs. 27) behave very similar in Co L-edge under oxidative conditions and that both Co_4_ and Co_27_ can be best described as CoO (in a good agreement with XANES) with the Co^2+^ cation in tetrahedral coordination. Since the spectra of Co_4_ and Co_27_ under the same experimental conditions are practically identical, in what follows we will discuss the Co_4_ case. Spectra in vacuum and under reaction condition display small but clearly discernable differences. In order to extract this information from the L-edge absorption spectra, the experimental UHV and ODH XAS spectra were simulated using the charge-transfer multiplet (CTM) approach^38–41^, (see Supplementary Figure 7 in Supplementary Information). The tetrahedral symmetry is chosen for the calculations, with the crystal field value 10Dq = −0.3 eV. The optical parameter *D*_t_ changes from −0.22 eV to −0.20 eV when simulating the UHV and the ODH spectrum, respectively. This subtle change in the *D*_t_ value indicates a slight change in the energy position of the d-orbitals^38^. The charge-transfer energy value (Δ) changes from 9 eV to 7 eV between the two states (UHV and ODH). A decrease in the Δ value corresponds to a decrease in the 3d^8^ to 3d^9^L ratio in the ground state, i.e. the 3d-state of Co interacts more with the delocalized electron from the oxygen 2p valence band. Therefore, this decrease in the Δ values implies that the covalent bonding between oxygen and cobalt becomes stronger during reaction.

### Insights into low-temperature activity

To understand the observed low-temperature activity of small Co clusters for cyclohexane oxidative dehydrogenation, we carried out DFT calculations on key steps for the reaction pathway involving the Co_4_ cluster. The DFT calculations were carried out for Co_4_O_4_ clusters based on the XANES results above showing that the Co is in a 2+ state. They were supported on a *θ*-alumina surface. The Co_4_O_4_ clusters should also be representative for the Co_27_ cluster. This can be justified by the similarities of the X-ray spectra of Co_4_ and Co_27_ showing a dominant CoO type composition, as well as the similar catalytic performance of the two clusters. The location of the cluster was optimized on the surface. As shown in the XANES studies the cluster remains oxidized throughout the experiment. Thus we assume that O_2_ cleavage is not a crucial step. The θ–alumina surface was chosen and is an approximation to the amorphous alumina surface created by ALD used in the experimental studies.

**Fig. 5:**
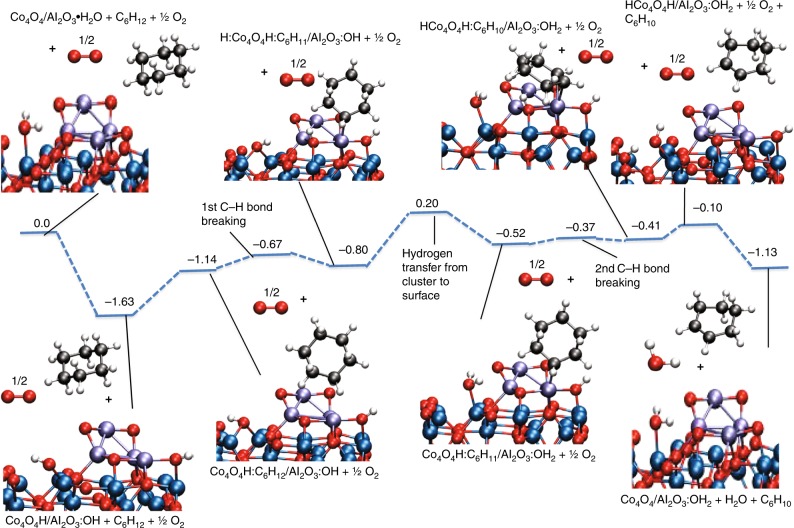
Key steps leading to the formation of cyclohexene from cyclohexane on the Co_4_O_4_ cluster. The values are ΔE + *S*_trans_ and are in eV

Within the temperature range studied, reaction pathway A (Fig. 1) appears as the predominant channel since cyclohexene is the major C_6_ species produced, moreover at the lowest temperature. The calculated transition states and intermediates for pathway A presented in Fig. 1, leading to formation of cyclohexene on the Co_4_O_4_ cluster are shown in Fig. 5. There is no apparent barrier (referenced to gas phase cyclohexane) for breaking of the first C–H bond and the true barrier for this step is 0.47 eV. The true barrier for breaking the second C–H bond is much smaller at 0.15 eV. The pathway is thermodynamically downhill by 0.41 eV to form cyclohexene, which binds to Co_4_O_4_ by its *π*-bond. The reason that the second barrier is lower than the first is that the presence of cyclohexyl group bound to cobalt weakens the Co–O bond and makes the site more active. The reason that the first and second C–H bond breaking barriers are low is the presence of hydrogen on the cluster that weakens the Co–O bond (makes it a dative bond as opposed to a covalent bond without hydrogen) destabilizing the cluster and making the Co–O site more active. Thus, the C–H bond interacts across the active Co–O decreasing the C–H activation energy. The hydrogen transfer to the alumina surface regenerates the Co–O active site for the breaking of the second C–H bond. The apparent barrier for the reaction is 0.20 eV corresponding to the hydrogen transfer. The hydrogens in this mechanism are assumed to initially come from the hydroxylated amorphous alumina surface via hydrogen transfer and subsequently from the ODH reaction. The subsequent steps involve the desorption of cyclohexene from the Co_4_ cluster and the regeneration of the catalyst by removal of the hydrogens through the oxygens. The pathway is completed by the regeneration of the catalyst by the removal of the hydrogen through hydroxyl groups on the surface. Thus, in the overall reaction scheme, surface hydroxyl groups serve as the means for removal of hydrogen from the catalyst as water. We also note that all atoms in the cluster play a role in the reaction pathway and that the reaction is not occurring at a single atom of the cluster.

## Discussion

The activation energy barrier determined from the experimental data for the Co_4_O_4_/Al_2_O_3_ catalyst for cyclohexene formation is 0.15 eV (as calculated from the Arrhenius plot shown in Supplementary Figure 9) and is a good agreement with the calculated value from DFT barrier of 0.20 eV shown in Fig. 5. The activation energy with Co clusters is substantially lower than the previously reported catalysts^29,42,43^. Theory leads to the conclusion that under-coordinated Co sites in small Co_n_O_m_ clusters are highly active for cyclohexane ODH. This can be explained by the attractive interaction between the under-coordinated Co and cyclohexane. The DFT calculations show that the initial adsorption complex between cyclohexane and the Co_4_O_4_ cluster (Fig. 5) results in significant charge transfer from a cyclohexane C–H bonding orbital to the cluster, as also shown in XAS simulations of the experimental spectra. The binding energy of cyclohexene to the cluster (0.6 eV, obtained relative to starting products) is especially notable since it is much lower than that of propene in the case of Pt clusters^8^. The weaker binding of cyclohexene could explain the lower temperature required for cyclohexane ODH compared to propane ODH on Pt clusters. The smaller binding energy is due to the oxidized nature of the Co clusters.

The work here reports on selective low temperature oxidative dehydrogenation of cyclohexane driven by subnanometer size oxidized cobalt clusters, with cyclohexene as the dominant product. Density functional studies explain that the high activity towards cyclohexene at low temperatures originates from the combination of the intrinsic reactivity of the small clusters combined with the small binding energy of cyclohexene on the oxidized clusters. This joint computational and experimental study indicate that cobalt-based clusters may hold a great promise for a new class of low-temperature oxidation catalysts for a broad spectrum of oxidative processes.

## Methods

### ALD alumina film preparation

The support, a ~3 ML thin alumina film was created by atomic layer deposition (ALD) on the top of a naturally oxidized n-type (phosphorus doped) Si chip. This ALD alumina support was proved to strongly bind subnanometer cobalt clusters with a binding energy of 3.2–4.6 eV^34^ and keep the clusters from sintering at elevated temperatures^30^.

### Deposition of size-selected clusters

The production of Co clusters was done using a well-established size-selective method in the gas phase, followed by soft-landing the clusters of desired size on the support^8,12^. Within this synthesis approach, the beam of positively charged Co clusters was produced in a vacuum apparatus in a laser vaporization cluster source utilizing the 532 nm wavelength radiation of a frequency doubled Nd:YAG laser focused on a rotating cobalt target rod. Helium was used as carrier gas, and the positively charged clusters led through an ion guide assembly made of an electrostatic lense assembly into a quadrupole mass filter. (Supplementary Figure 1 shows typical mass spectra of positively charged cobalt clusters with the throughput of the cluster apparatus tuned for smaller or larger cobalt clusters.) Next, the Co_4_ or Co_27_ clusters of interest were selected out of the beam on the quadrupole mass filter and deflected towards the support using an electrostatic quadrupole deflector. Finally, the Co_4_ or Co_27_ clusters were deposited with a kinetic energy of less than 1 eV/atom on the alumina support. In order to preserve the size specificity of the clusters on the support upon landing, the applied surface coverage was limited to 0.1 of an atomic monolayer equivalent (ML). The amount of deposited metal and the level of surface coverage were determined by real-time monitoring of the flux of clusters landing on the support and the diameter of the area covered by clusters. At the given surface coverage, the average inter-cluster distance is estimated to be within ~2–4 nm, as also verified by transmission electron microscopy (TEM) collected on other cluster samples with similar surface coverage^44^. After deposition, the cluster samples are exposed to air, which may cause the oxidation of cobalt.

### Combined in situ GISAXS, GIXANES, and TPRx

experiments under a pressure of 800 mbar was used to monitor the sintering resistance, oxidation state, and catalytic performance at the advanced photon source facility (APS, Sector 12-ID-C) of Argonne National Laboratory. The reactant mixture comprised of cyclohexane (0.4%) in helium which was pre-mixed with oxygen to attain a 1:10 (or 10:1) cyclohexane to oxygen ratio and was fed into the reactor at a flow of 30 sccm^29^. Details of the GISAXS/GIXANES/TPRx approach are given for example in ref. ^45^. The reaction products were analyzed using a differentially pumped mass-spectrometer (Pfeiffer Vacuum Prisma QMS 200) continuously sampling from the reaction cell. Typical raw reactivity data are shown in Supplementary Figure 10. The rates of formation of products were calculated using the number of deposited Co atoms and calibrating the mass spectrometer using diluted calibrated gas mixtures (AirGas). The GIXANES data were analyzed with IFEFFIT interactive software package (with ATHENA and ARTEMIS graphical interfaces)^46^. The GISAXS data were collected on a Platinum detector developed by the Advanced Photon Source (1024 × 1024 pixels) with X-rays of 7.6 keV as a function of temperature. The two-dimensional GISAXS images were then cut both in horizontal and vertical directions. The scattering is then compared with background on the alumina thin film.

### In situ XAS characterization

Additional characterization was performed at the BESSY II photon source (Helmholtz-Zentrum Berlin) using in situ TEY (total electron yield) X-ray absorption (XAS) on the Co L-edge under 1 mbar pressure, combined with product analysis using a microGC (Varian). Details about the experimental setup can be found for example in ref. ^47^. The XAS experiments were performed on identical Co_4_ and Co_27_ samples as those used at the APS, under ultra-high vacuum (UHV) and in situ conditions under 1 mbar pressure with 12 sccm gas flow of a gas mixture containing 96% He, 2% cyclohexane, and 2% oxygen.

### Simulations of the XAS spectra

The experimental XAS spectra were simulated using the charge-transfer multiplet (CTM) approach^38–41^. The calculations have been carried out using the CTM4XAS vs3.1 program^48^. The difference between the core hole potential and the 3d–3d repulsion energy as well as the hopping parameters were taken from literature data^40,41^. Only the L_3_ edge is presented in the experimental results, since the L_3_ to L_2_ ratio is practically the same in the experimental and simulated curves. The core hole potential *Q* is taken 2.0 eV higher than the 3d–3d repulsion energy *U*_dd_. The value for the hopping *e*_g_ electrons (*t*_e_) is 1.0 eV, while for the hopping *t*_2g_ electrons (*t*_t_) is 2.0 eV.

### Density functional theory

All calculations used periodic DFT as implemented in the Vienna Ab initio Simulation Package 5.2 (VASP)^49,50^. The ion–electron interactions were described using the projector augmented wave (PAW) potentials with an energy cutoff of 400 eV for the plane wave basis set. The generalized gradient corrected Perdew–Burke–Ernzerhof (GGA-PBE) functional was used for all periodic calculations with Monkhorst-Pack 5 × 5 × 1 k-point sampling^51,52^. Optimizations were carried out until the forces converged to 0.02 eV/Å for geometry optimizations and 0.05 eV/Å for nudged elastic band calculations using the quasi-Newton method for geometry optimization. The Co_4_O_4_ cluster was supported on a θ-Al_2_O_3_ surface which was modeled in a slab geometry with a (2 × 2) lateral periodicity. Six repeated Al_2_O_3_ layers were used to describe the surface, of which the three top layers were allowed to relax. The location and structure of the Co_4_O_4_ cluster was optimized on the surface  see Supplementary Figure 8). The slabs are separated by a vacuum distance of 16 Å. The cluster has a magnetic moment and therefore spin- polarization was considered in all calculations. Zero-point energies are not included.

## Supplementary information

Supplementary Information

## Data Availability

The datasets generated during and/or analysed during the current study are available from the corresponding authors on reasonable request.
