# Supplementary material for: Subnanometer cobalt oxide clusters as selective low temperature oxidative dehydrogenation catalysts
Source: Nat Commun. 2019 Feb 27;10:954. doi: 10.1038/s41467-019-08819-5 (PMC6393513; doi:10.1038/s41467-019-08819-5)
Supplement: Supplementary file 1 — Supplementary Information [file 41467_2019_8819_MOESM1_ESM.pdf]

SUPPLEMENTARY INFORMATION

Subnanometer Cobalt Oxide Clusters as Selective Low Temperature Oxidative Dehydrogenation Catalysts

Lee *et al.*

Content

Supplementary Figures

Supplementary Figure 1| Typical mass spectra of cationic Co clusters generated in the cluster source.

Supplementary Figure 2| XANES spectra of supported Co_4 and Co_{27} clusters, oxygen rich conditions (C_6H_{12}:O_2=1:10)

Supplementary Figure 3| XANES spectra of supported Co_4 and Co_{27} clusters, oxygen lean conditions (C_6H_{12}:O_2=10:1)

Supplementary Figure 4| Typical fits of XANES spectra, oxygen lean conditions (C_6H_{12}:O_2=10:1)

Supplementary Figure 5| Typical fits of XANES spectra, oxygen rich conditions (C_6H_{12}:O_2=1:10)

Supplementary Figure 6| Co $L_{3,2}$-edge NEXAFS absorption spectra.

Supplementary Figure 7| Co $L_{3,2}$ -edge reference NEXAFS spectra.

Supplementary Figure 8| Model structure of alumina-supported Co_4O_4.

Supplementary Figure 9| Arrhenius plots.

Supplementary Figure 10| Typical raw TPRx mass spectrometry data.

Supplementary Tables

Supplementary Table 1: Formation rates for products (*r*) obtained at BESSY

Supplementary Table 2: Formation rates for products (*r*) obtained at ANL/APS

Supplementary Table 3: Reported performance of catalysts for C_6H_6 production

Supplementary References

Supplementary Figures

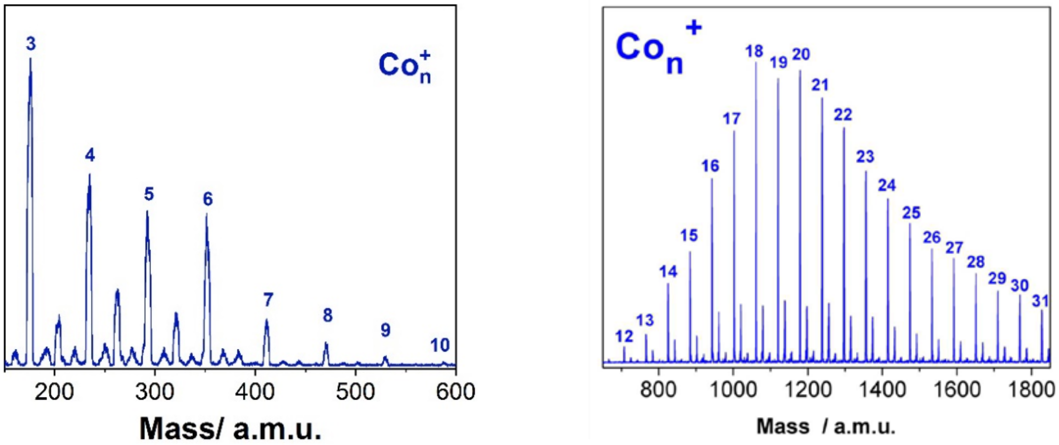

Supplementary Figure 1| Typical mass spectra of cationic Co clusters generated in the cluster source. Left: Throughput of the cluster apparatus optimized for ~3-10 atom clusters. Right: Throughput optimized for ~14-31 atom clusters. (The two mass spectra were recorded at different mass resolution.) The pairs of minor peeks following the Co cluster peaks correspond to clusters with 1 and 2 oxygen atoms from the oxygen traces present.).

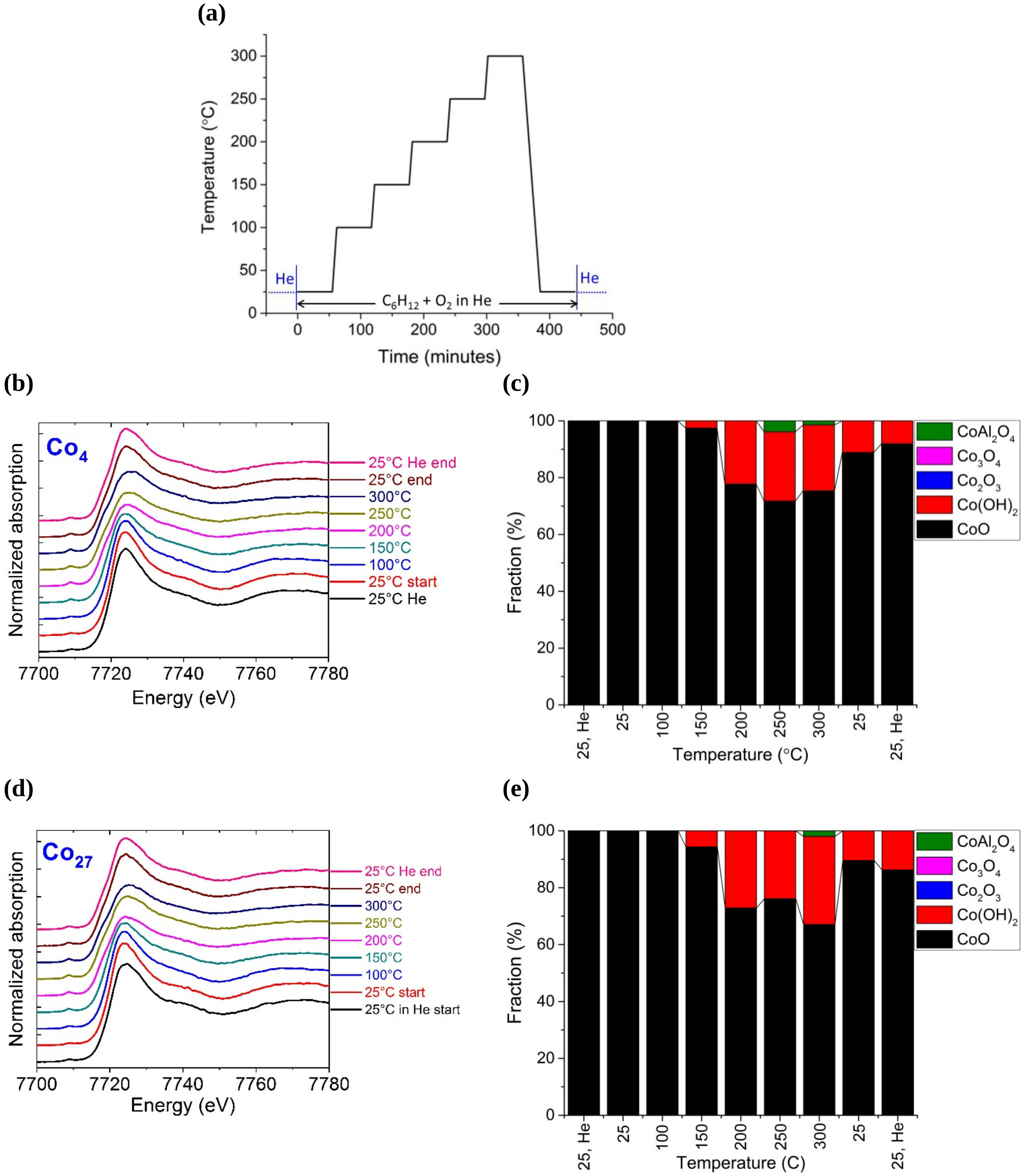

Supplementary Figure 2| XANES spectra of supported Co_4 and Co_{27} clusters, oxygen rich conditions (C_6H_{12}:O_2=1:10). **(a)** Temperature ramp with gas environment applied. **(b)** and **(c)** Co K-edge XANES spectra of Co_4/Al_2O_3 and results from linear combination fit of the spectra using bulk standards CoO, Co_2O_3, Co_3O_4, $Co(OH)_2$, and $CoAl_2O_4$ and Co_{27}, respectively. **(d)** and **(e)** Co K-edge XANES spectra of Co_{27}/Al_2O_3 and results from linear combination fit of the spectra using bulk standards. CoO, Co_2O_3, Co_3O_4, $Co(OH)_2$, and $CoAl_2O_4$ and Co_{27}, respectively. The XANES spectra of the bulk standards are presented in Figure 4 of the main text. The contribution from CoOOH was zero (not shown).

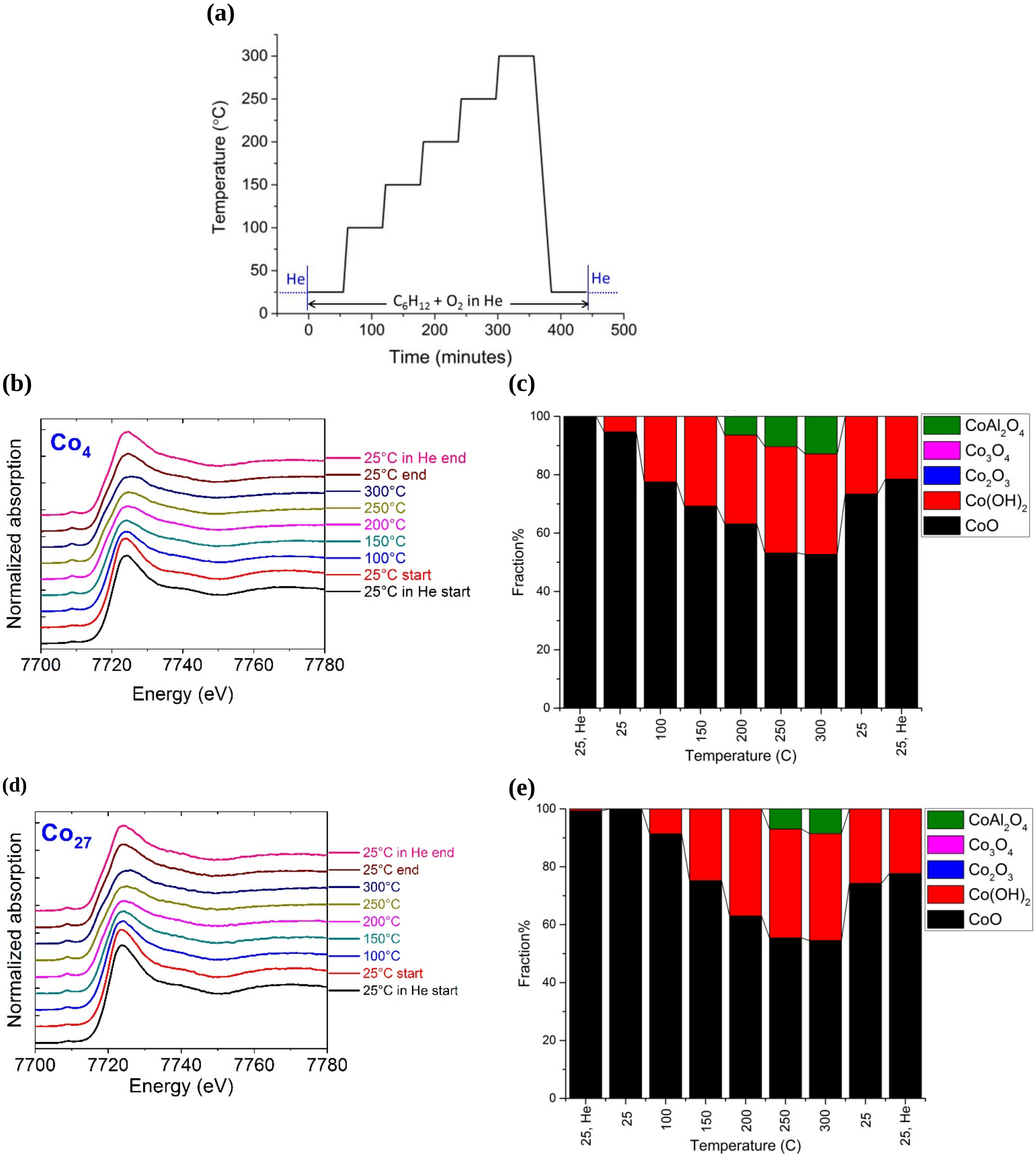

Supplementary Figure 3| XANES spectra of supported Co_4 and Co_{27} clusters, oxygen lean conditions (C_6H_{12}:O_2=10:1). **(a)** Temperature ramp with gas environment applied. **(b)** and **(c)** Co K-edge XANES spectra of Co_4/Al_2O_3 and results from linear combination fit of the spectra using bulk standards CoO, Co_2O_3, Co_3O_4, $Co(OH)_2$, and $CoAl_2O_4$ and Co_{27}, respectively. **(d)** and **(e) Co K-edge** XANES spectra of Co_{27}/Al_2O_3 and results from linear combination fit of the spectra using bulk standards. CoO, Co_2O_3, Co_3O_4, $Co(OH)_2$, and $CoAl_2O_4$ and Co_{27}, respectively. The XANES spectra of the bulk standards are presented in **Figure 4** of the main text. The contribution from CoOOH was zero (not shown).

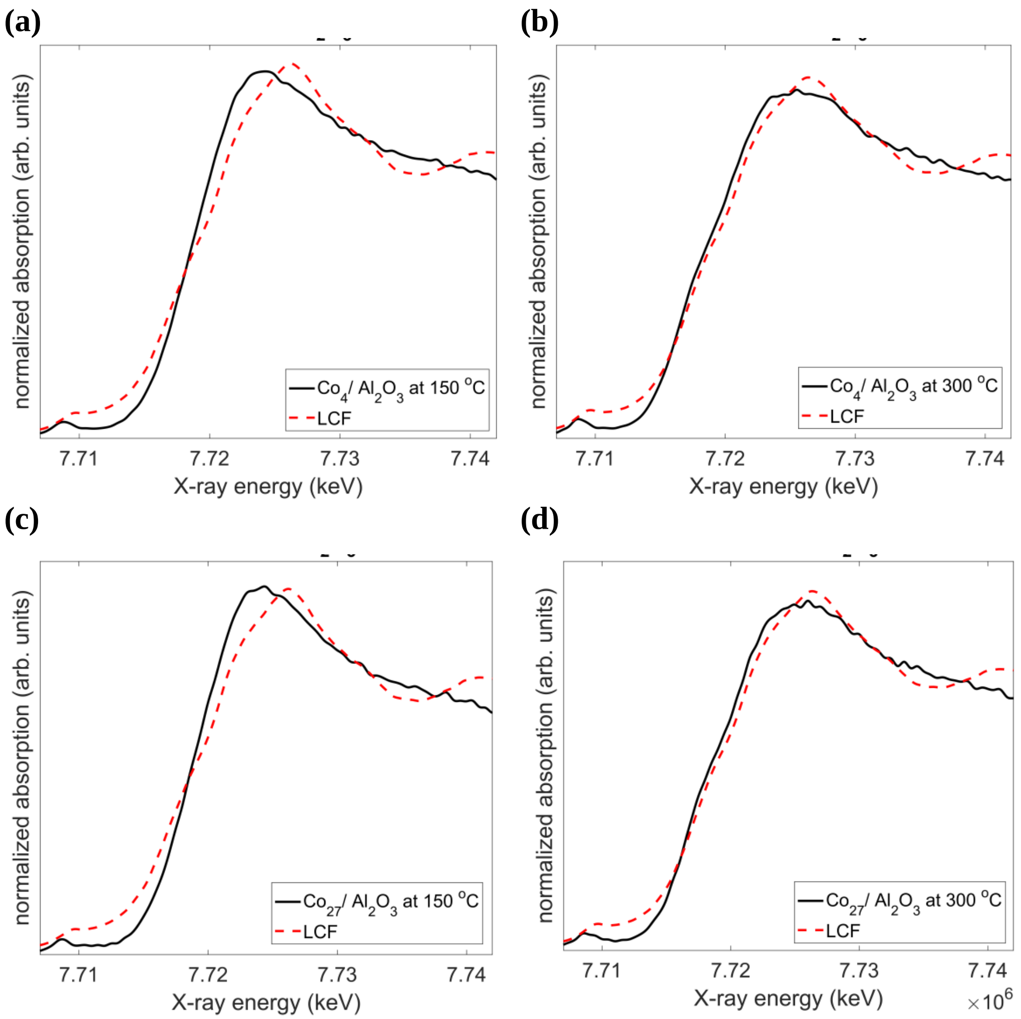

Supplementary Figure 4| Typical fits of XANES spectra, oxygen lean conditions (C_6H_{12}:O_2=10:1) The raw and fitted spectra are plotted in black and red color respectively. **(a)** Co_4/Al_2O_3 at 150 ˚C, **(b)** Co_4/Al_2O_3 at 300 ˚C, **(c)** Co_{27}/Al_2O_3 at 150 ˚C, and **(d)** Co_{27}/Al_2O_3 at 300 ˚C.

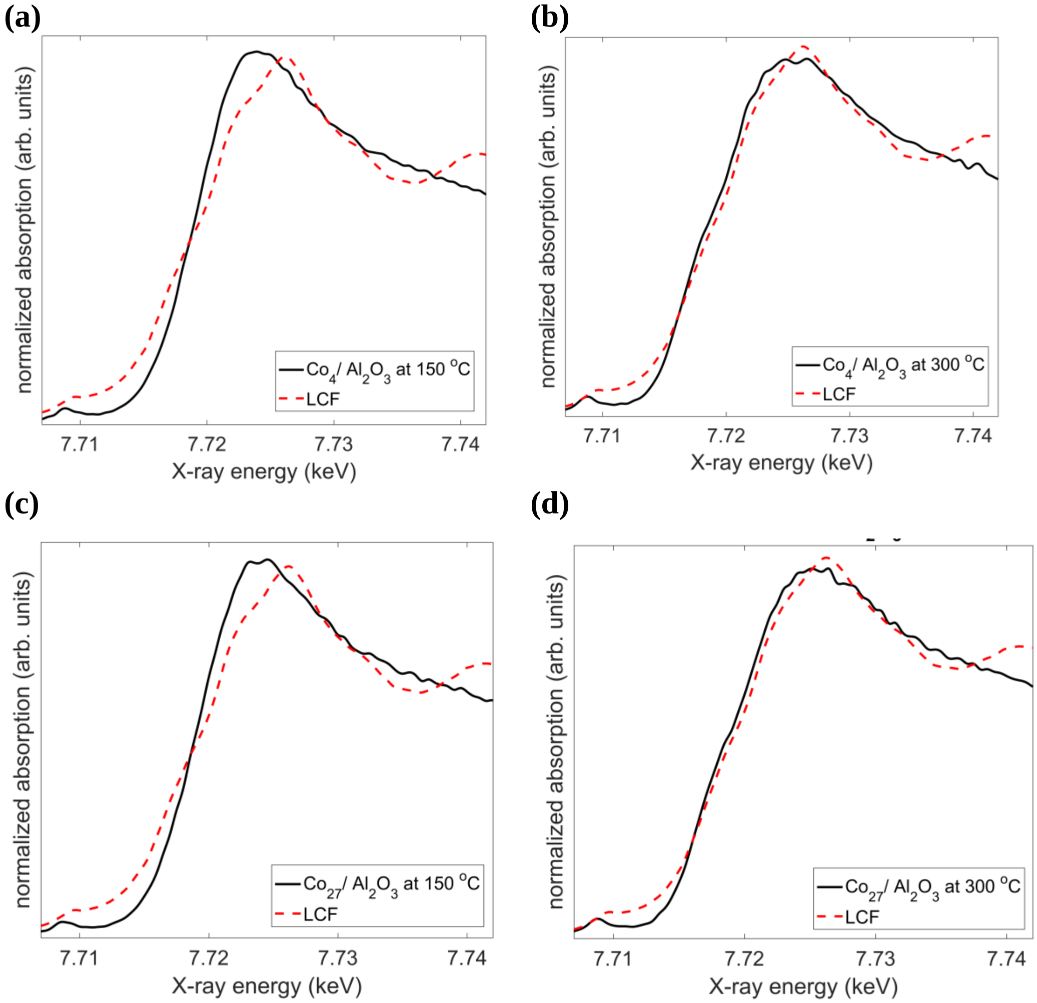

Supplementary Figure 5| Typical fits of XANES spectra, oxygen rich conditions (C_6H_{12}:O_2=1:10). The raw and fitted spectra are plotted in black and red color respectively. **(a)** Co_4/Al_2O_3 at 150 ˚C, **(b)** Co_4/Al_2O_3 at 300 ˚C, **(c)** Co_{27}/Al_2O_3 at 150 ˚C, and **(d)** Co_{27}/Al_2O_3 at 300 ˚C.

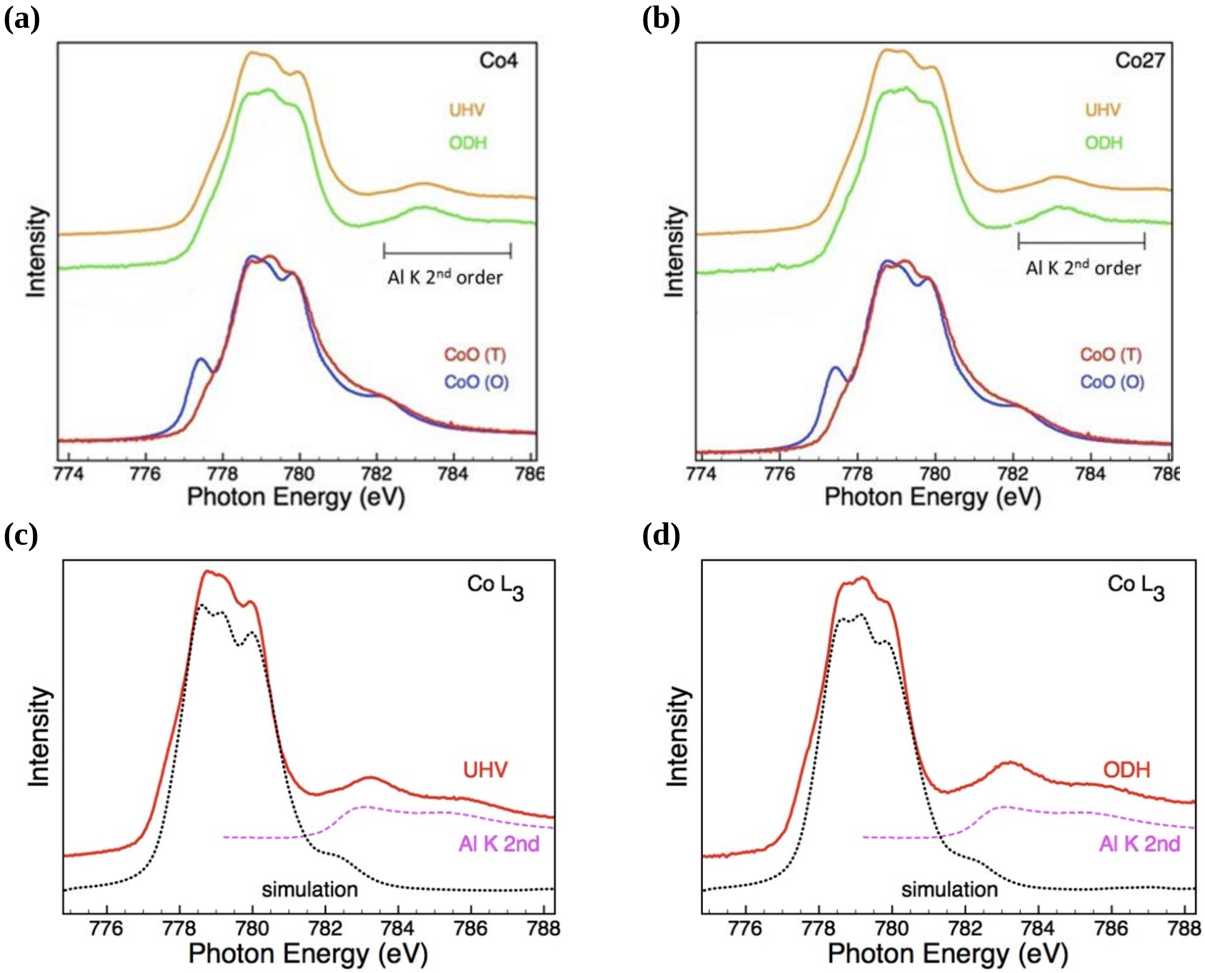

Supplementary Figure 6| Co $L_{3,2}$-edge NEXAFS absorption spectra. Co L_3-edge spectra of **(a)** Co_4/Al_2O_3 and **(b)** Co_{27}/Al_2O_3 clusters on alumina under UHV at RT, and in cyclohexane oxidative dehydrogenation (Co_4/Al_2O_3 at 220 ºC and Co_{27}/Al_2O_3at 250 ºC) are shown, along with the reference CoO in octahedral and tetrahedral Co coordination. **(c)** Co_4/Al_2O_3 under UHV and theoretically simulated curve of tetrahedral Co^{2+} cations (D_t = -0.22 eV, Δ= 9 eV).; **(d)** Co_4/Al_2O_3 under oxidative dehydrogenation of cyclohexane and theoretically simulated curve of tetrahedral Co^{2+} cations (D_t = -0.20 eV, Δ= 7 eV). Note the experimental Co L-edge spectrum overlaps with the second order Al K-edge absorption spectrum of the substrate.

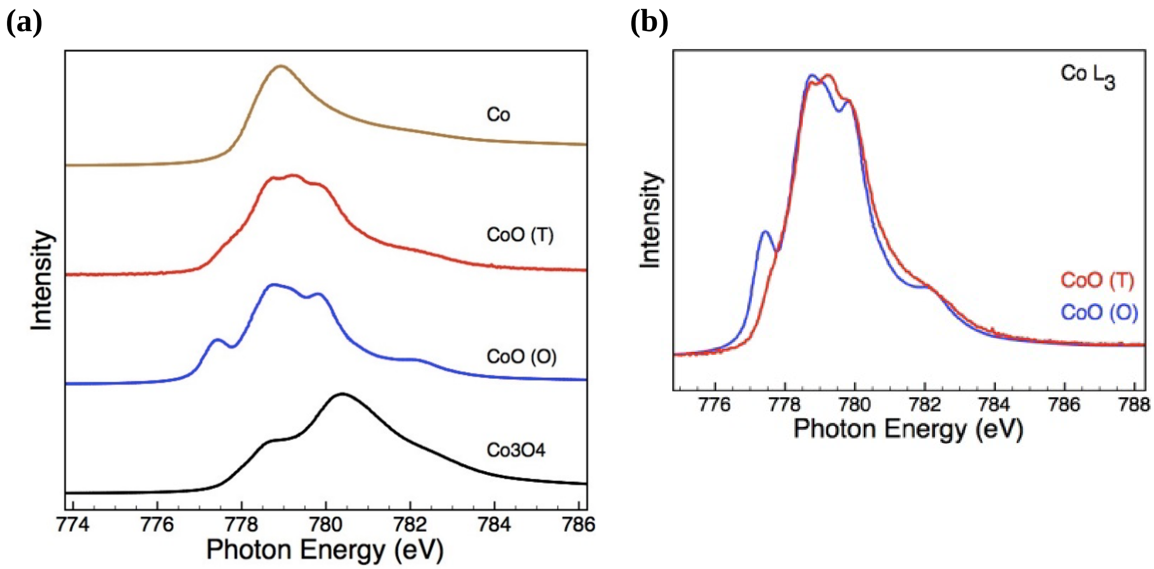

Supplementary Figure 7| Co $L_{3,2}$ -edge reference NEXAFS spectra. (a) reference spectra of metallic Co, Co_3O_4, CoO in octahedral and CoO tetrahedral Co coordination. **(b)** Overlapped spectra of CoO in octahedral and tetrahedral Co coordination.

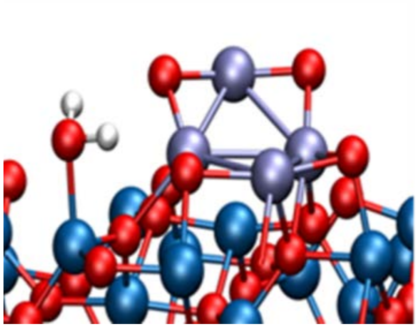

Supplementary Figure 8| **Model structure of alumina-supported Co_4O_4.**

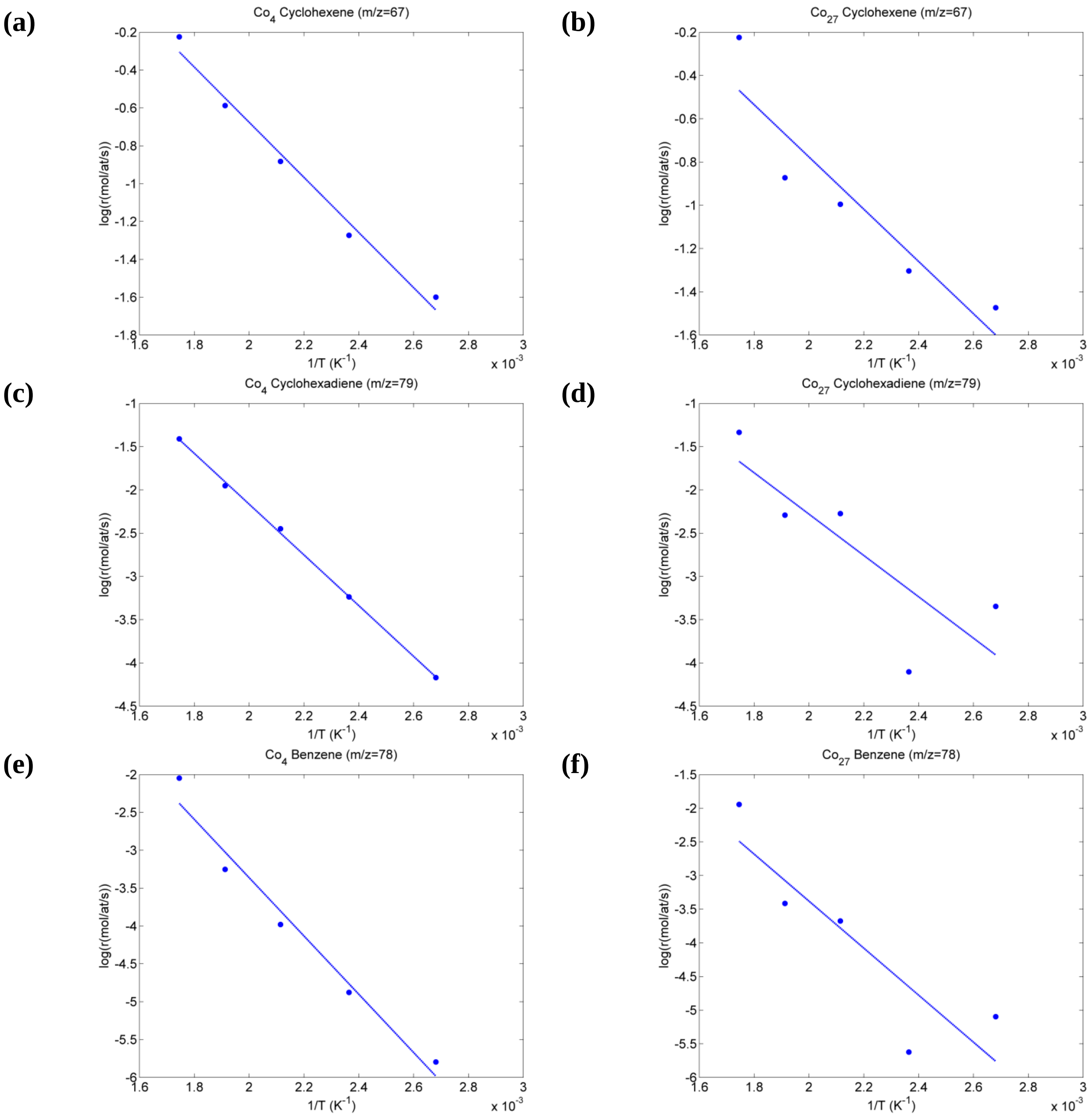

Supplementary Figure 9: Arrhenius plots for **(a)** Co_4 (m/z=67; Cyclohexene), **(b)** Co_4 (m/z=79; Cyclohexadiene), **(c)** Co_4 (m/z=78; Benzene), **(d)** Co_{27} (m/z=67; Cyclohexene), **(e)** Co_{27} (m/z=79; Cyclohexadiene), **(f)** Co_{27} (m/z=78; Benzene)

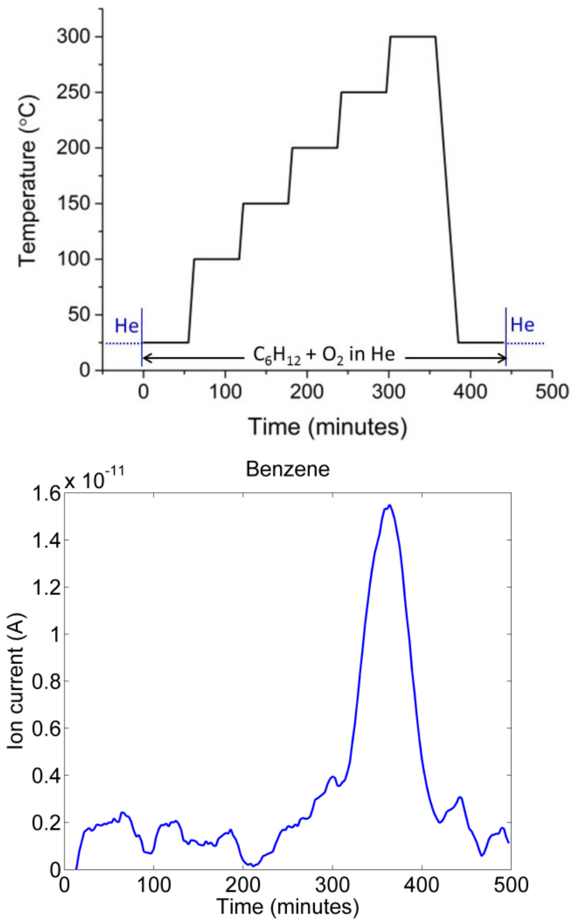

Supplementary Figure 10| Typical raw TPRx mass spectrometry data. Bottom: Time-dependent evolution of the m/z= 78 signal used to monitor the benzene, for the Co_4 cluster sample at under oxygen rich conditions (C_6H_{12}:O_2=1:10). Top: Applied temperature ramp.

Supplementary Tables

Supplementary Table 1: Formation rates for products (*r*) measured under a gas mixture at pressure of 1 mbar.

		r [mol produced / Co mol / s]				
**Catalyst**	**T [ºC]**	**C_6H_{10}**	**1,3-C_6H_8**	**1,4-C_6H_8**	**1,3 C_6H_8 + 1,4 C_6H_8**	**C_6H_6**
**Co_4/Al_2O_3**	180	0.064	0.26	0.019	0.279	0.019
**Co_4/Al_2O_3**	220	0.15	0.5	0.025	0.525	0.025
**Co_{27}/Al_2O_3**	180		0.077		0.077	
**Co_{27}/Al_2O_3**	250	0.11	0.086		0.086	0.027

Gas flow conditions: 12 ml/min flow of 96% He, 2% cyclohexane and 2% oxygen

Supplementary Table 2: Formation rates for products (*r*) obtained at ANL/APS.

**Cluster**	**C_6H_{12}: O_2**	**Temperature (°C)**	**C_6H_{10} *r* molecules/ atom/s**	**C_6H_8 *r* molecules/ atom/s**	**C_6H_6 *r* molecules/ atom/s**
Co_4	1:10	100	0.31	0.07	0.01
		150	0.44	0.08	0.01
		200	0.66	0.12	0.03
		250	0.88	0.17	0.05
		300	1.14	0.22	0.13
Co_{27}	1:10	100	0.22	0.05	0.01
		150	0.27	0.05	0.01
		200	0.38	0.08	0.02
		250	0.44	0.08	0.03
		300	0.64	0.14	0.08
Co_4	10:1	100	.04	0.02	0.01
		150	0.07	0.05	0.01
		200	0.1	0.05	0.01
		250	0.1	0.07	0.02
		300	0.12	0.08	0.04
Co_{27}	10:1	100	0.06	0.04	0.01
		150	0.08	0.04	0.01
		200	0.07	0.03	0.01
		250	0.09	0.08	0.06
		300	0.15	0.13	0.14

Supplementary Table 3: Reported performance of catalysts for C_6H_6 production. * denotes TOFs and *r*s reported in the papers, the other values were calculated based on data provided in the paper and estimated number of surface/total atoms.

Sample	Cluster size	Metal loading	T (°C)	TOR (C_6H_6/ CO_2) molecules/ total atom/s	*r* (C_6H_6/ CO_2) molecules/surf. atom or active site/s
TiO_2 [1]	≈ 10 nm		400	0.0006	
5% Au/ TiO_2 [1]	≈ 10 nm	0.05 g	400	0.0014	
5% Pd/ TiO_2 [1]	≈ 10 nm	0.05 g	200<br>400	0.0010<br>0.0020	
2.5% Au 2.5% Pd/ TiO_2 [1]	≈ 10 nm	0.05 g	400	0.0014	
Co_3O_4 [2]	6 nm	19.4 µg	225<br>300	$2.42 \cdot 10^{-5}$/ 0.00371*<br>0.0137/ 0.0505	$3.33 \cdot 10^{-5}$/ 0.00676*<br>0.0188/ 0.0689
Co_3O_4 [2]	12 nm	15.4 µg	225<br>300	$4.15 \cdot 10^{-5}$/ 0.00034*<br>0.0026/ 0.00568	$1.75 \cdot 10^{-4}$/ 0.00142*<br>0.0110/ 0.0238
FeOx octahedra [3]	≈ 20 nm	10 mg	350<br>400	0.00028/ 0.0005<br>0.00035/ 0.0007	14.38/ 25.44<br>18.25/ 37.06
sAu/ Fe_3O_4 octahedra[3]	≈ 20 nm	10 mg	350<br>400	0.00046/ 0.0001<br>0.00035/ 0.0003	26.38/ 6.68<br>19.70/ 14.36
FeO_x cubes[3]	≈ 20 nm	10 mg	350<br>400	0.00012/ 0.0003<br>0.00006/ 0.0003	1.66/ 4.52<br>0.86/ 4.45
Au/FeO_x cubes (parent)[3]	≈ 20 nm	10 mg	350<br>400	0.00077/ 0.0005<br>0.00011/ 0.0009	13.69/ 9.20<br>2.04/ 15.94
Au/ FeO_x cubes (NaCN leached)[3]	≈ 20 nm	10 mg	350<br>400	0.00004/ 0.0012<br>0.00003/ 0.0012	0.72/ 22.02<br>0.48/ 22.26
Au/ FeO_x cubes (NaCN leached, reduced in 5% H_2 at 200 °C) [3]	≈ 20 nm	10 mg	350<br>400	0.00039/ 0.0003<br>0.00014/ 0.0003	6.96/ 5.46<br>2.64/ 6.02
VO_x monoliths [4]		0.20 mg	450	0.0071/0.0020	0.01
MoO_3/ Al_2O_3, Unsupported MOVS<br>Supported MOVS [5]	15–50 nm<br>1-15 µm		400	$6.65 \cdot 10^{-6}$ / -<br>$20.48 \cdot 10^{-6}$ / -	
Al,Cr15/5 [6]		5 mg	320		0.0032*
NiO_2/ γ-Al_2O_3 [7]		0.2 g	300		0.0012*
$CoTiO_3$ on TiO_2 [8]		0.2 g	400	0.0017 / -	
0.14-L_2Cu_6[9]<br>(multinuclear copper siloxide cluster)		0.1 g	250<br>350		0.0000552*<br>0.0007776

Supplementary References

1 Dummer, N. F., Bawaked, S., Hayward, J., Jenkins, R. & Hutchings, G. J. Reprint of: Oxidative dehydrogenation of cyclohexane and cyclohexene over supported gold, -palladium catalysts. *Catal. Today* **160**, 50-54, doi:10.1016/j.cattod.2010.12.014 (2011).

2 Tyo, E. C. *et al.* Oxidative Dehydrogenation of Cyclohexane on Cobalt Oxide (Co3O4) Nanoparticles: The Effect of Particle Size on Activity and Selectivity. *ACS Catal.* **2**, 2409-2423, doi:10.1021/cs300479a (2012).

3 Goergen, S. *et al.* Structure Sensitivity of Oxidative Dehydrogenation of Cyclohexane over FeOx and Au/Fe3O4 Nanocrystals. *ACS Catal.* **3**, 529-539, doi:10.1021/cs3007582 (2013).

4 Feng, H., Elam, J. W., Libera, J. A., Pellin, M. J. & Stair, P. C. Oxidative dehydrogenation of cyclohexane over alumina-supported vanadium oxide nanoliths. *J. Catal.* **269**, 421-431, doi:10.1016/j.jcat.2009.11.026 (2010).

5 Alyea, E. C. & Keane, M. A. The oxidative dehydrogenation of cyclohexane and cyclohexene over unsupported and supported molybdena catalysts prepared by metal oxide vapor deposition. *J. Catal.* **164**, 28-35, doi:10.1006/jcat.1996.0359 (1996).

6 Lezanska, M., Szymanski, G. S., Pietrzyk, P., Sojka, Z. & Lercher, J. A. Characterization of Cr-MCM-41 and Al,Cr-MCM-41 mesoporous catalysts for gas-phase oxidative dehydrogenation of cyclohexane. *J Phys Chem C* **111**, 1830-1839, doi:10.1021/jp066498u (2007).

7 AbdelDayem, H. M., Faiz, M., Abdel-Samad, H. S. & Hassan, S. A. Rare earth oxides doped NiO/gamma-Al2O3 catalyst for oxidative dehydrogenation of cyclohexane. *J. Rare Earth.* **33**, 611-618, doi:10.1016/S1002-0721(14)60461-0 (2015).

8 Sarkar, B., Pendem, C., Konathala, L. N. S., Sasaki, T. & Bal, R. Formation of ilmenite-type CoTiO3 on TiO2 and its performance in oxidative dehydrogenation of cyclohexane with molecular oxygen. *Catal. Commun.* **56**, 5-10, doi:10.1016/j.catcom.2014.06.021 (2014).

9 Nauert, S. L., Schax, F., Limberg, C. & Notestein, J. M. Cyclohexane oxidative dehydrogenation over copper oxide catalysts. *J. Catal.* **341**, 180-190, doi:10.1016/j.jcat.2016.07.002 (2016).
